# Compact Planar Ultrawideband Antennas with 3.5/5.2/5.8 GHz Triple Band-Notched Characteristics for Internet of Things Applications

**DOI:** 10.3390/s17020349

**Published:** 2017-02-10

**Authors:** Jian Dong, Qianqian Li, Lianwen Deng

**Affiliations:** 1 School of Information Science and Engineering, Central South University, Changsha 410083, China; liqianqian@csu.edu.cn; 2 School of Physics and Electronics, Central South University, Changsha 410083, China; denglw@csu.edu.cn

**Keywords:** ultrawideband (UWB) antenna, band-notched, slots, Wireless Local Area Network (WLAN), Worldwide Interoperability for Microwave Access (WiMAX)

## Abstract

Ultrawideband (UWB) antennas, as core devices in high-speed wireless communication, are widely applied to mobile handsets, wireless sensor networks, and Internet of Things (IoT). A compact printed monopole antenna for UWB applications with triple band-notched characteristics is proposed in this paper. The antenna has a very compact size of 10 × 16 mm^2^ and is composed of a square slotted radiation patch and a narrow rectangular ground plane on the back of the substrate. First, by etching a pair of inverted T-shaped slots at the bottom of the radiation patch, one notched band at 5–6 GHz for rejecting the Wireless Local Area Network (WLAN) is generated. Then, by cutting a comb-shaped slot on the top of the radiation patch, a second notched band for rejecting 3.5 GHz Worldwide Interoperability for Microwave Access (WiMAX) is obtained. Further, by cutting a pair of rectangular slots and a C-shaped slot as well as adding a pair of small square parasitic patches at the center of the radiating patch, two separate notched bands for rejecting 5.2 GHz lower WLAN and 5.8 GHz upper WLAN are realized, respectively. Additionally, by integrating the slotted radiation patch with the narrow rectangular ground plane, an enhanced impedance bandwidth can be achieved, especially at the higher band. The antenna consists of linear symmetrical sections only and is easy for fabrication and fine-tuning. The measured results show that the designed antenna provides a wide impedance bandwidth of 150% from 2.12 to 14.80 GHz for VSWR < 2, except for three notched bands of 3.36–4.16, 4.92–5.36, and 5.68–6.0 GHz. Additionally, the antenna exhibits nearly omnidirectional radiation characteristics, low gain at the stopbands, and flat group delay over the whole UWB except at the stopbands. Simulated and experimental results show that the proposed antenna can provide good frequency-domain and time-domain performances at desired UWB frequencies and be an attractive candidate for portable IoT applications.

## 1. Introduction

Since the Federal Communication Commission (FCC) allowed its commercial ultrawideband (UWB) systems to work from 3.1 GHz to 10.6 GHz in 2002 [1,2], UWB technology has attracted much attention because of broad bandwidth, good radiation characteristics, high speed data rate, and so on [3,4]. UWB antennas have found their niche in applications involving high or low data rate transmission over short ranges [5], surveillance systems [6], medical applications [7,8], wireless body area network (WBAN) [9], and Internet of Things (IoT) [10]. Compact and low power-consumption antennas are considered essential for portable WBAN or IoT sensors [11,12,13] as they can be easily embedded within these devices to reduce their complexity and/or weight. However, due to extremely wide working bands for UWB systems, the collision of UWB communication systems and some narrow communication systems is inevitable, such as Worldwide Interoperability for Microwave Access (WiMAX) systems operating at 3.3–3.7 GHz and Wireless Local Area Network (WLAN) systems operating at 5.15–5.35 GHz and 5.725–5.825 GHz [14]. Hence, in order to avoid potential interferences from narrow communication systems, it is desirable to design miniaturized UWB antenna with intrinsic band-notched characteristics at those narrow bands [15].

The concept of UWB antennas with frequency band rejected function was firstly proposed in [16,17], and various methods for designing band-notched UWB antennas were then reported, including adding parasitic resonators or tuning stubs [18,19,20,21] and etching slots [22,23,24,25,26,27,28,29]. In [22,23,24], a single band-notched function was realized for suppressing interferences from the WLAN bands (5.15–5.825 GHz). In order to avoid the interference from both the WiMAX (3.3–3.69 GHz) and WLAN (5.15–5.825 GHz) bands, dual band-notched UWB antennas were presented in [18,19,20] and [25,26,27,28,29]. However, most of these antennas have the common defect of a relatively large size, which may pose a challenge to the miniaturization antenna design for mobile device applications. Moreover, these band-notched designs for rejecting the WLAN bands completely rejected the whole 5–6 GHz frequency band, although the desired notched bands are 0.2 GHz (5.15–5.35 GHz) for lower WLAN bands and 0.1 GHz (5.725–5.825 GHz) for upper WLAN bands. Thus, any useful information included at 5.35–5.725 GHz will also be lost, meaning degraded received information and a lower signal quality.

Thus, UWB antennas with triple-notched bands are highly appreciated for rejecting lower and upper WLAN bands along with the WiMAX band. It is difficult to design triple band-notched UWB antennas due to the difficult bandwidth control of notched bands in a very limited space and complicated couplings between the adjacent notched bands. In [21], by using double open-circuited stubs at left and right edges of the radiating patch, a sharp rejection of both lower and upper WLAN bands was achieved. In [30], by using a hook-shaped defected ground structure and etching slots on the radiation patch as well as adding a ring on the back of the antenna, triple-notched frequency bands at 3.3–3.9, 5.2–5.35, and 5.8–6.0 GHz were achieved. In [31], by using a pair of slots and a complementary split ring resonator (CSRR) on the radiation patch and a pair of CSRRs on the ground plane, triple-notched frequency bands at 3.4–3.6, 5.1–5.3, and 5.7–5.9 GHz were achieved. In [32], by etching one CSSR inside a circular exciting stub on the front side and employing an interrogation-shaped defected ground structure as well as symmetrically adding a pair of open-circuit stubs, triple-notched frequency bands at 3.3–4, 5.15–5.4, and 5.8–6.1 GHz were achieved. In [33], by embedding two C-shaped slots on the radiation patch and a pair of symmetric C-shaped slots on the ground, triple-notched frequency bands at 3.43–3.65, 4.95–5.25, and 5.36–5.85 GHz were obtained. In [34], by embedding a reversed F-shaped slot in the patch and a reversed U-shaped slot in the feedline as well as adding a parasitic flipped C-shaped strip around the patch, triple-notched frequency bands at 3.20–4.19, 5.02–5.32, and 5.51–6.10 GHz were obtained. In [35], by using three complementary co-directional split ring resonator (SRRs), triple-notched frequency bands centered at 3.4, 5.25, and 5.78 GHz were obtained. In [36], by adding three capacitively loaded loop (CLL) resonators close to the feed line, triple-notched frequency bands at 3.29–3.67, 5.12–5.35, 5.67–5.83 GHz were achieved. Despite their realization of triple band-notched function, these designs have some drawbacks, such as large overall size, complicated irregular structures (e.g., the combination of CSSR, stubs, and slots), and incomplete rejecting for the WLAN and WiMAX bands.

In this paper, a compact planar UWB antenna with 3.5/5.2/5.8 GHz triple-notched bands is presented. First, by etching a pair of inverted T-shaped slots at the bottom of the radiating patch, a notched band at 5–6 GHz for rejecting WLAN is created. Then, a second notched band for rejecting 3.5 GHz WiMAX is obtained by embedding a comb-shaped slot on the top of the radiating patch. Further, by cutting a pair of rectangular slots and a C-shaped slot as well as adding a pair of small square parasitic patches at the center of the radiating patch, the first notched band at 5–6 GHz are divided into two separate notched bands for rejecting 5.2 GHz lower WLAN and 5.8 GHz upper WLAN, respectively. By integrating the slotted radiating patch with a narrow rectangular ground plane, an enhanced impedance bandwidth can be achieved, especially at the higher band. The proposed antenna consists of linear symmetrical sections only and has a much smaller size and a wider operation bandwidth than most of the recently reported band-notched UWB antennas [18,19,20,21,22,23,24,25,26,27,28,29,30,31,32,33,34,35,36]. Simulated and measured results show that our proposed antenna provides good frequency-domain and time-domain performances in the frequency band of operation. Detailed analysis of the proposed antenna is given below.

## 2. Antenna Design 

**Figure 1:**
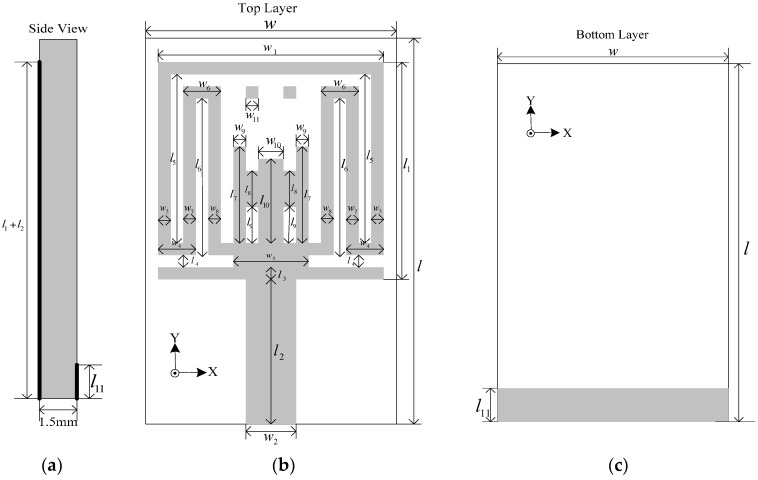
Geometry of the proposed antenna: (**a**) side view; (**b**) top view; (**c**) bottom view.

Figure 1 presents the schematic geometry of the proposed antenna. This antenna is printed on 1.5-mm-thick FR4 substrates with a relative permittivity of 4.4 and a loss tangent of 0.02, while the overall areas are only 10 × 16 mm^2^. The basic antenna structure is composed of a square radiation patch, a microstrip feedline, and a ground plane. The width of the microstrip feedline is set to be 2 mm to achieve 50 Ω characteristic impedance. A narrow rectangular ground plane with dimensions of 10 × 1.5 mm^2^ is placed on the back of the substrate. The structure of the radiation patch is symmetrical with respect to the vertical direction, and the size of the patch is 9 × 9 mm^2^. By embedding a pair of inverted T-shaped slots, a comb-shaped slot, and a pair of rectangular slots and a C-shaped slot as well as adding a pair of square parasitic patches, three notched bands centered at 3.5, 5.2, and 5.8 GHz are generated. Our final antenna design is obtained by adjusting the dimensions of the radiation patch, the slots and ground plane. All the parameters are optimized by Ansoft electromagnetic simulator HFSS and specified in Table 1. Detailed analysis of the proposed antenna will be presented from the following three aspects.

### 2.1. Design Principle

**Figure 2:**
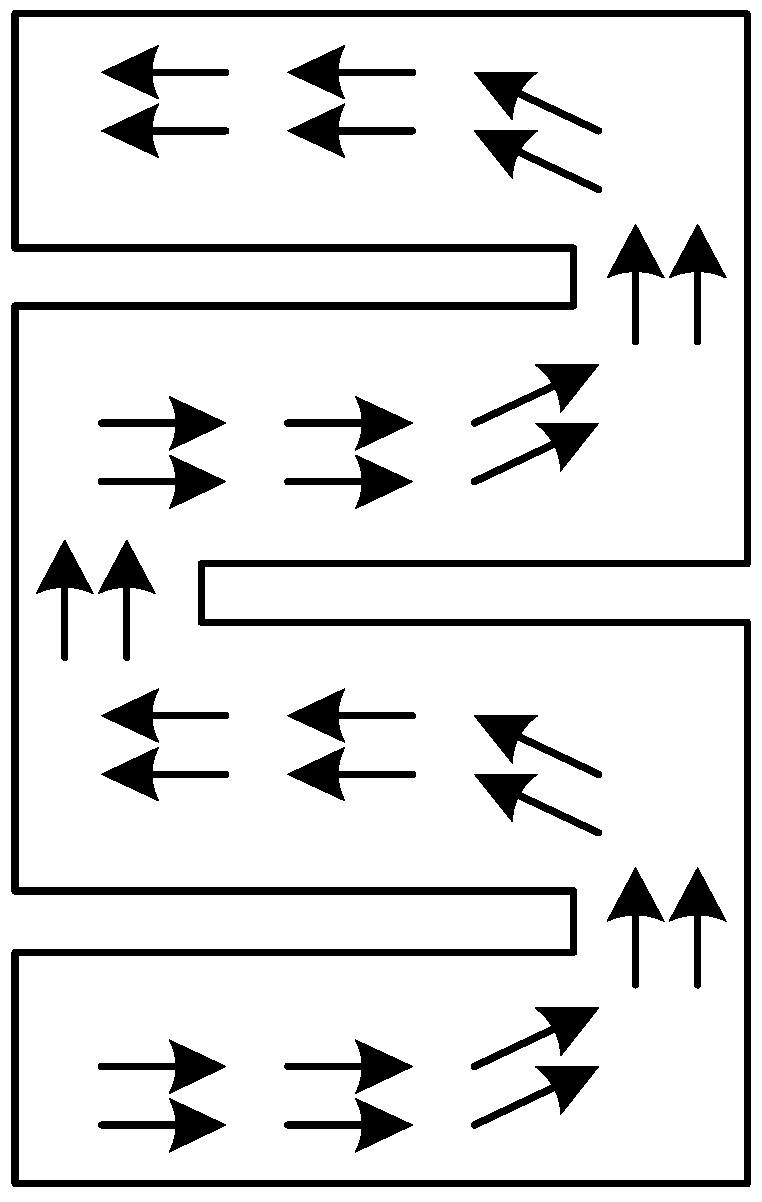
Schematic diagram of surface current distribution on rectangular patch with slots.

Etching slots is one of the most efficient techniques in designing miniaturized multi-band-notched UWB antenna as it can be easily realized without any additional cost of size or expense. Slots with various shapes are often used in band-notched antenna designs, e.g., etching L-shaped, F-shaped, C-shaped, U-shaped, and arc-shaped slots on the radiation patch or on the ground plane [22,23,24,25,26,27,28,29,30,31,32,33,34]. Cutting slots changes the surface current distribution on the radiation patch or ground and increases the effective current path length (see in Figure 2). Subsequently, the size of the antenna will be significantly reduced for realizing a given resonant frequency. Additionally, the bandwidth of the antenna will be broadened due to the decreased Q value caused by etching slots.

By properly adjusting dimensions of the slot resonators, the band-notched UWB antenna can provide flexible tuning of the bandwidth with desirable band notched function. As a first order of approximation, the total length of a slot resonator can be selected to be a half guide wavelength according to the following expression:

(1) $$L_{t}=\frac{\lambda_{g}}{2}$$

where *λ_g_* is the wavelength in the medium and can be calculated by [29,37,38]:

(2) $$\frac{\lambda_{g}}{\lambda_{0}}=A(W,h,\varepsilon_{r})-B(W,h,\varepsilon_{r},\lambda_{0})\ln(\frac{h}{\lambda_{0}})$$

where

(3) $$A=0.9217-0.277\ln(\varepsilon_{r})+0.0322(\frac{W}{h}){\left[\frac{\varepsilon_{r}}{W/h+0.435}\right]}^{1/2}$$

(4) $$B=0.046-\frac{0.0365}{\varepsilon_{r}^{2}\sqrt{W/\lambda_{0}}(9.06-100W/\lambda_{0})}$$

and *λ*_0_ = *c*/*f*_0_ is the wavelength in the free space, *c* is the free-space velocity of light, and *f_0_* is resonator frequency; *ε_r_* is the relative permittivity of the substrate, *W* is the width of a slot line, and *h* is the substrate thickness. This equation is valid for 3.8 ≤ *ε_r_* ≤ 9.8, 0.0015 ≤ *W/λ*_0_ ≤ 0.075.

Using Equations (1) and (2), we can obtain the following equation which predicts the length of the half-wavelength resonator in terms of *W*, *h*, *ε_r_*, and the notch frequency *f*_0_:

(5) $$L_{t}(W,h,\varepsilon_{r},f_{0})=\frac{c}{2f_{0}}\left[A(W,h,\varepsilon_{r})-B(W,h,\varepsilon_{r},f_{0})\ln(\frac{f_{0}h}{c})\right]$$

Assuming the center frequency *f*_0_ of the WLAN notched band is 5.5 GHz, and *W* = 0.5 mm, *h* = 1.5 mm, *ε_r_* = 4.4, and the total length of the two inverted T-shaped slots is computed to be 18.9 mm. The practical total length of the two inverted T-shaped slots optimized by EM simulator HFSS is 19 mm. Additionally, the length of the comb-shaped slot can be estimated in a similar way.

### 2.2. Structural Analysis

**Figure 3:**
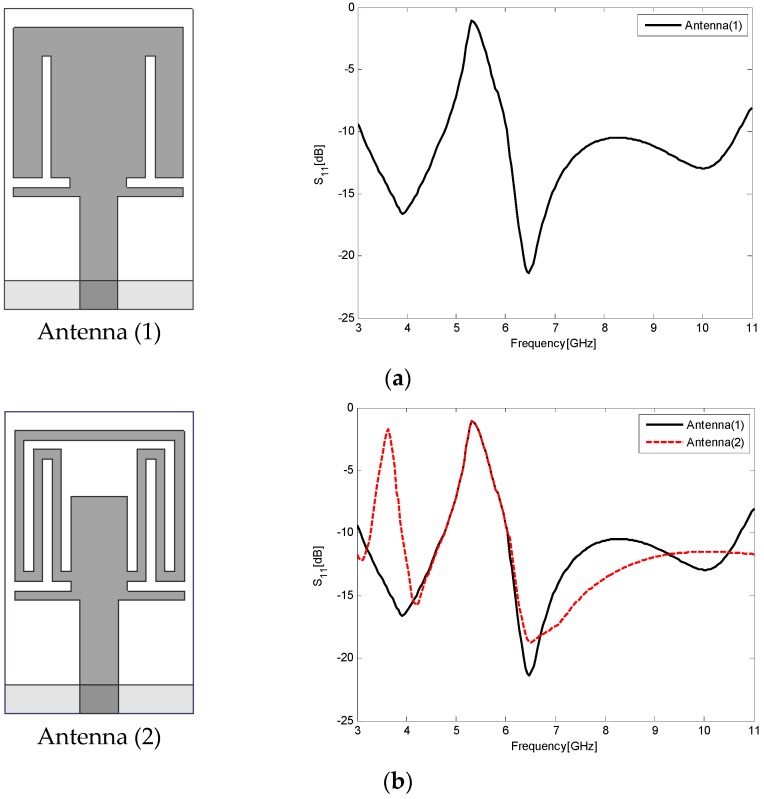

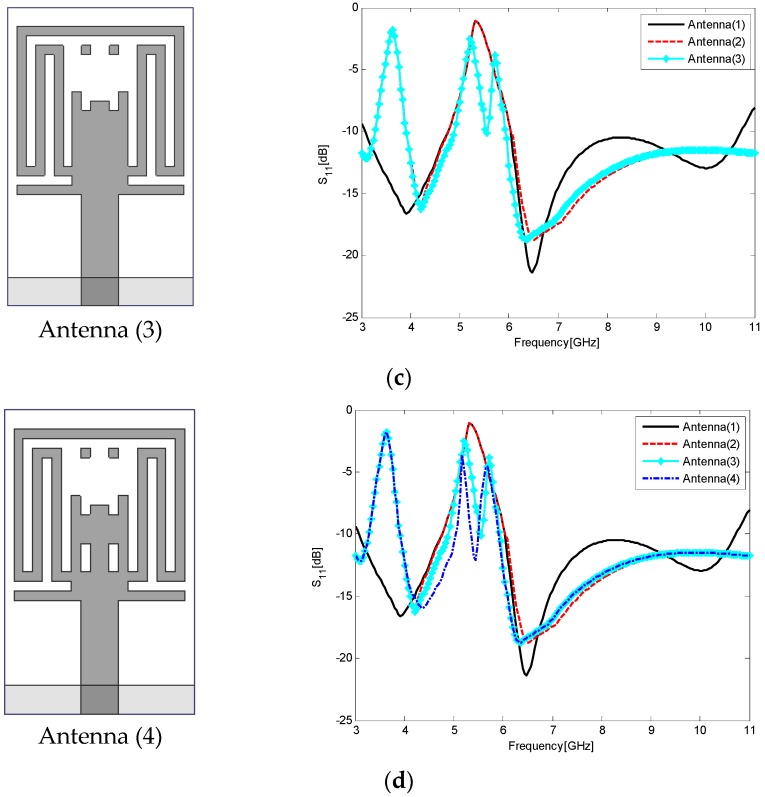
The step-by-step evolution process of the proposed antenna and the corresponding S_11_ results at different stages. The left column gives the geometries of various antennas involved in the design evolution process. The right column gives the simulated S_11_ results for the various antenna geometries. (**a**) Step 1; (**b**) Step 2; (**c**) Step 3; (**d**) Step 4.

To clearly illustrate how the triple band-notched antenna was designed, Figure 3 shows the step-by-step evolution process of the proposed antenna and the corresponding S_11_ results at different stages. First, in order to obtain single band-notched function for rejecting WLAN (5.15–5.825 GHz), we embed a pair of inverted T-shaped slots at the bottom of the radiation patch (Antenna (1)). Then, by etching a comb-shaped slot on the top of the radiation patch, a second notched band rejecting the 3.5 GHz WiMAX is generated (Antenna (2)). Finally, in order to divide the whole 5–6 GHz notched band into two separate notched bands for rejecting 5.2 GHz lower WLAN and 5.8 GHz upper WLAN, respectively, we cut a C-shaped slot and add a pair of small square parasitic patches (Antenna (3)) firstly and then cut a pair of rectangular slots (Antenna (4)) at the center of the radiation patch.

**Figure 4:**
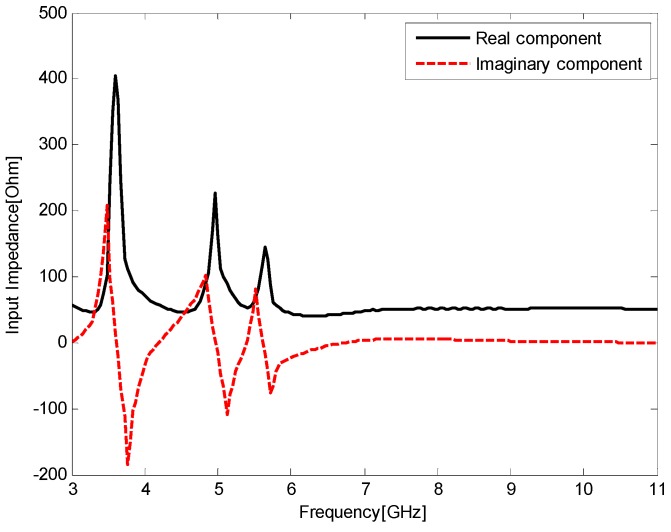
Input impedance *Z* of the antenna versus frequency.

The input impedance *Z_in_* of the antenna versus frequency is given in Figure 4. When the antenna is operating at the passbands, the input resistance is around 50 Ω and the input reactance is around 0 Ω, indicating that the proposed antenna is suitable for UWB applications. At the stopbands, their values largely deviate from the nominal values, indicating obvious impedance mismatch at these frequencies. 

**Figure 5:**
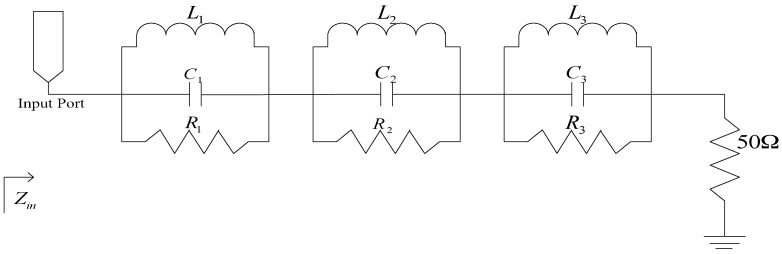
Schematic of the equivalent circuit model of the triple band-notched ultrawideband (UWB) antenna.

As shown in Figure 4, the antenna input impedance at the reject bands is similar to that of a lumped parallel RLC circuit. Therefore, the proposed antenna can be modeled as three parallel RLC resonators connected in series with a 50 Ω load as shown in Figure 5. In that figure, the first parallel RLC resonates at 3.5 GHz and a maximum input impedance *Z_in_* is observed. Thus, a total impedance mismatch occurs between the feed line and the radiating patch, and the first band-notched characteristic is generated. Similarly, the second parallel RLC resonates at 5.2 GHz and the third at 5.8 GHz, while the load approximates the radiation resistance of the antenna. The input resistance of the equivalent circuit at the notched bands is maximum while its input reactance almost vanishes. Essentially, the triple band-notched function is achieved by these resonators.

**Figure 6:**
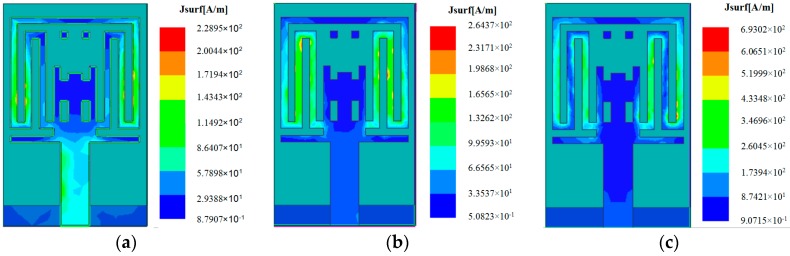
Simulated surface current distributions on the radiation patch for the proposed antenna at (**a**) 3.5 GHz; (**b**) 5.2 GHz; and (**c**) 5.8 GHz.

In order to further explain the triple band-notched function resulting from slotted structures, the simulated surface current distributions for the proposed antenna at the notched frequencies of 3.5, 5.2, and 5.8 GHz are shown in Figure 6. It is clearly observed from the figure that the current distributions are different at the three notched bands. Specifically, as shown in Figure 6a, the currents are mainly concentrated on the edge of the comb-shaped slot at the lower notched band (i.e., 3.5 GHz WiMAX band), as shown in Figure 6b,c, the currents are mainly distributed around the invert T-shaped slots at the higher notched bands (i.e., 5.2 GHz lower WLAN and 5.8 GHz upper WLAN bands). Therefore, the antenna impedance mismatch occurs at these frequencies due to the band-notched functions of these slotted structures.

### 2.3. Parametric Study

Due to the miniaturized size, the reflection performance of the proposed antenna is sensitive to the variation of geometrical parameters. To illustrate the effects of the critical parameters on the notched bands, a parametric study on the proposed antenna is given below. 

**Figure 7:**
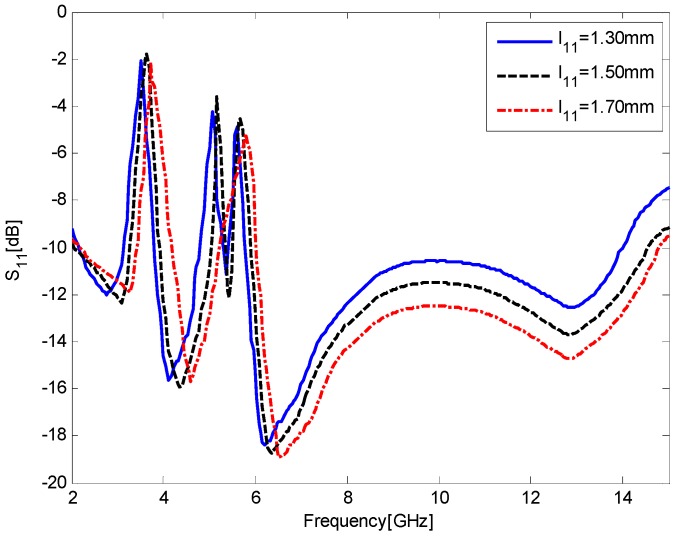
Simulated S_11_ results for the antenna as a function of *l*_11_, the height of the ground plane.

Keeping all the other parameters fixed, the effect of *l*_11_, the height of the ground plane, on the return loss is depicted in Figure 7. As illustrated in Figure 7, the value of the ground height plays an important role in the broadband impedance matching and band-notched characteristics because it can adjust the electromagnetic couplings between the radiation patch and the ground plane. Specifically, the S_11_ curve shifts toward higher frequency as *l*_11_ increases from 1.3 mm to 1.7 mm. When *l*_11_ = 1.7 mm, the antenna exhibits only a dual band-notched function. Considering the coverage of notched bands and wider impedance bandwidth at the higher band, the value of *l*_11_ = 1.5 mm is chosen as an optimum.

**Figure 8:**
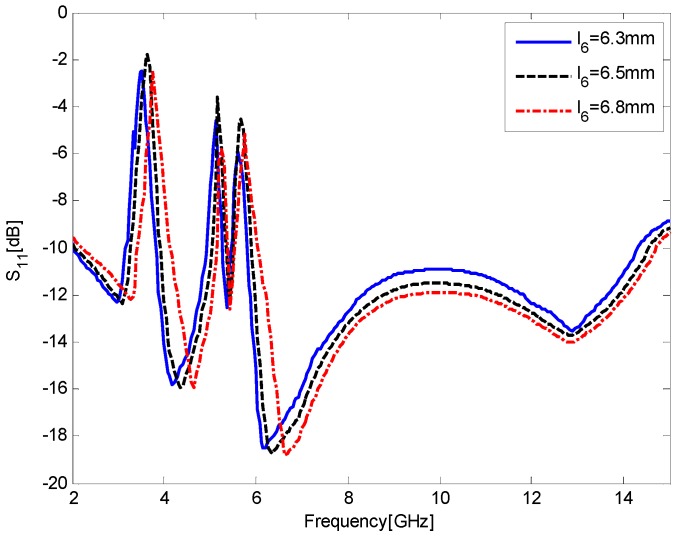
Simulated S_11_ results for the antenna as a function of *l*_6_, the vertical length of the inverted T-slots.

Figure 8 shows the simulated S_11_ results for the antenna as a function of *l*_6_, the vertical length of the inverted T-slots. All the curves have triple-notched bands, but the curve of *l*_6_ = 6.3 mm has a relatively weaker band-notched capability at the second and third bands, and the curve of *l*_6_ = 6.8 mm cannot completely reject the 3.5 GHz WiMAX bands (3.3–3.7 GHz). Thus, the value of *l*_6_ = 6.5 mm is chosen as an optimum. 

**Figure 9:**
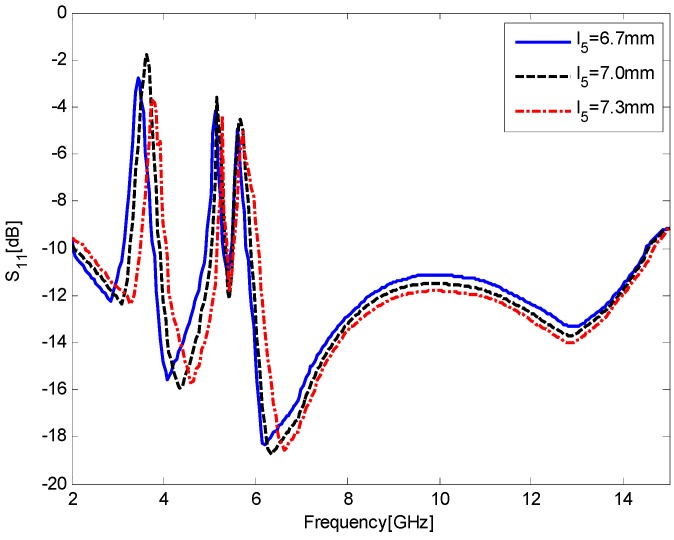
Simulated S_11_ results for the antenna as a function of *l*_5_, the vertical length of the comb-shaped slot.

Figure 9 shows the simulated S_11_ results for the antenna as a function of *l*_5_, the vertical length of the comb-shaped slot. Similarly, all the curves exhibit triple-notched bands, but the curve of *l*_5_ = 6.7 mm has a relatively weaker band-notched capability at the first band, and the curve of *l*_5_ = 7.3 mm cannot completely reject the 3.5 GHz WiMAX bands (3.3–3.7 GHz). Thus, the optimum value of *l*_5_ is chosen as 7.0 mm.

## 3. Experimental Results and Discussions

### 3.1. Frequency-Domain Performance

**Figure 10:**
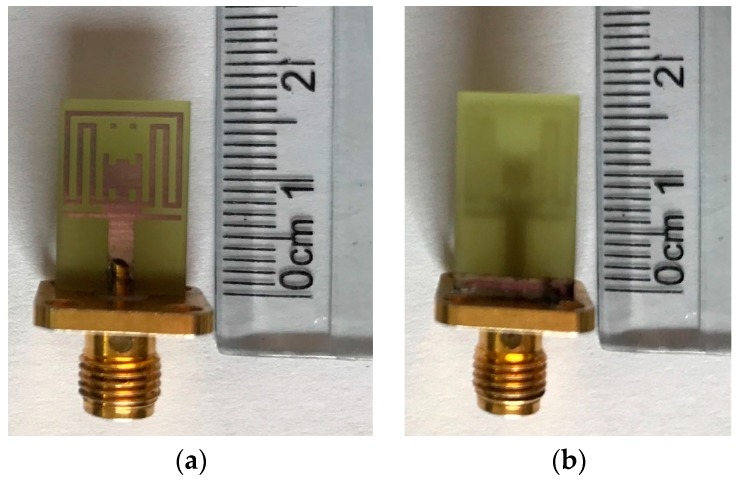
Photos of the fabricated triple band-notched UWB antenna: (**a**) front view; (**b**) back view.

**Figure 11:**
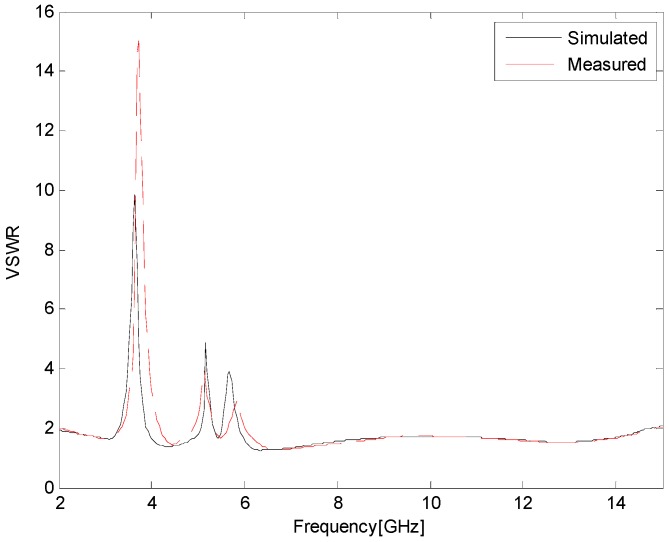
Simulated and measured VSWR of the proposed triple band-notched UWB antenna.

The prototype antenna with the optimal dimensions listed in Table 1 was fabricated and tested, as shown in Figure 10. Figure 11 presents the simulated and measured VSWR of the proposed antenna. The measured results are obtained by using an Anritsu 37347D vector network analyzer (40 MHz–20 GHz, Anritsu Corporation, Atsugi, Kanagawa, Japan). It can be seen that the simulated impedance bandwidth for VSWR <2 is 2.20–14.68 GHz, covering the entire UWB frequency band, except for triple-notched bands of 3.32–3.88, 5.0–5.36, and 5.52–5.92 GHz. The maximum simulated VSWR values in the triple-notched bands are 9.8 (at 3.64 GHz), 4.9 (at 5.16 GHz), and 3.9 (at 5.68 GHz), respectively. At the same time, the measured impedance bandwidth for VSWR <2 is 2.12–14.80 GHz, except for 3.36–4.16, 4.92–5.36, and 5.68–6.0 GHz. The maximum measured VSWR values in the triple-notched bands are 15.1 (at 3.68 GHz), 4.0 (at 5.12 GHz), and 3.0 (at 5.84 GHz), respectively. Good agreement between the simulated and measured results is observed. The small discrepancy between them may be caused by fabrication tolerance and the introduction of the SMA connector.

**Figure 12:**
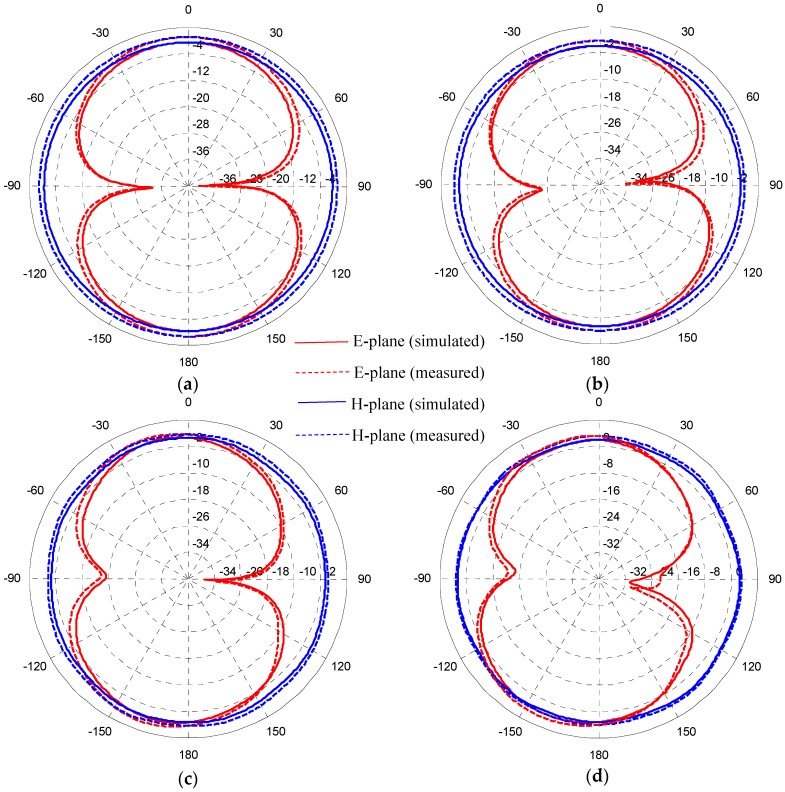
Simulated and measured radiation patterns for the proposed triple band-notched UWB antenna at (**a**) 3 GHz; (**b**) 4.5 GHz; (**c**) 8 GHz; (**d**) 12 GHz.

In order to illustrate that the proposed antenna can actually radiate over a very wide frequency band, Figure 12 presents the simulated and measured radiation patterns in the E-plane (yz-plane) and the H-plane (xz-plane) at 3, 4.5, 8, and 12 GHz, respectively, showing a good agreement between simulated and measured results. The antenna was tested in an anechoic chamber with dimensions 7.6 m × 5.5 m × 3.8 m. A double ridge horn antenna was used as a reference antenna, and the fabricated antenna was placed on a turntable with a diameter of 1 m face to face with respect to the reference antenna. During measurement, the prototype antenna was rotated with a rotation angle of 360° spaced by 1°. It is observed that the proposed antenna provides fairly good omnidirectional H-plane patterns and bidirectional monopole-like E-plane patterns over four different passbands. Small distortions in the E-plane and H-plane patterns begin to occur at higher frequencies because the radiating elements are no longer small enough relative to those wavelengths.

**Figure 13:**
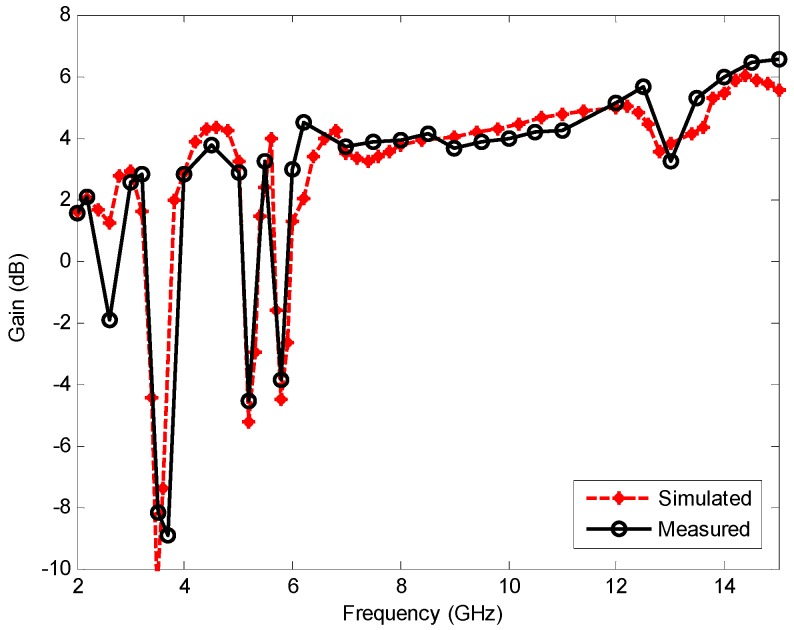
Simulated and measured gain for the proposed triple band-notched UWB antenna.

Figure 13 shows the simulated and measured gain variations with the frequency for the proposed antenna, indicating a good agreement between simulated and measured results. Three sharp decreases of about −12, −9, and −8 dB in the notched bands at 3.5, 5.2, and 5.8 GHz are observed, which clearly confirms the signal-rejection capability of the proposed antenna. The sharp gain reductions in the notched bands can be expected from Figure 11 because high VSWR values in these frequencies lead to low total efficiency and hence low gain. At other frequencies outside the notched bands, the antenna gain increases with the frequency and varies from 2 to above 6 dB. This means that the proposed antenna can ensure the quality of UWB communication links in WLAN and WiMAX environments by filtering coexisting narrowband interferences at practically no additional fabrication cost.

### 3.2. Time-Domain Performance

As shown in the previous section, the proposed antenna presents good frequency-domain performance. However, as for UWB antenna, good frequency-domain performance cannot necessarily ensure that the antenna also behaves well in the time domain. Some multiresonant antennas (e.g., Yagi-Uda and log-periodic antennas) may seriously widen the narrow pulse in the time domain due to multiple reflections in their structures [39]. Therefore, to evaluate the suitability of the proposed antenna for pulse-based UWB communication systems, it is necessary to investigate its time-domain characteristics, including the transmission coefficient, group delay, and pulse waveform analysis. 

**Figure 14:**
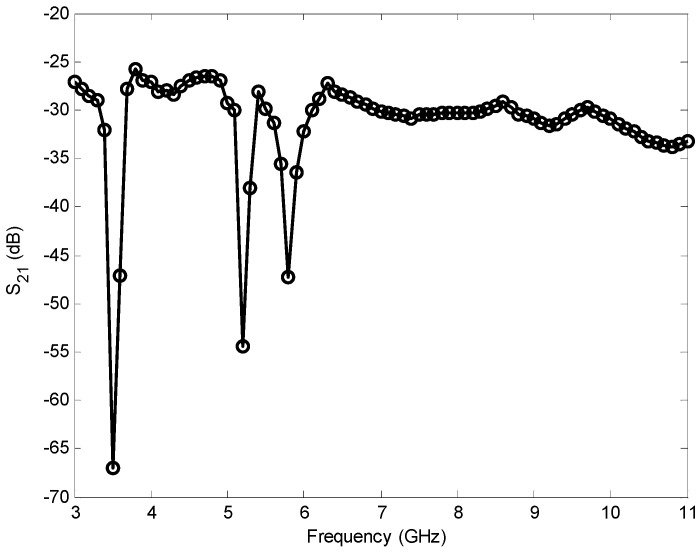
Transmission coefficient of the proposed antenna.

The transmission coefficient (|S_21_|) and group delay were measured using two identical proposed antenna facing each other and separated by a distance of 20 cm, allowing far field conditions at all UWB frequencies. The measured coefficient is shown in Figure 14. Three sharp decreases (about 35, 25, and 20 dB) of the transmission coefficient in the notched bands at 3.5, 5.2, and 5.8 GHz were observed. These sharp reductions can be interpreted from the results of VSWR and gain because large reflection coefficients in these frequencies lead to low gain and hence low transmission magnitude response. At other frequencies outside the notched bands, the antenna exhibits approximately flat magnitude of transmission response, which indicates stable UWB transmission capability.

**Figure 15:**
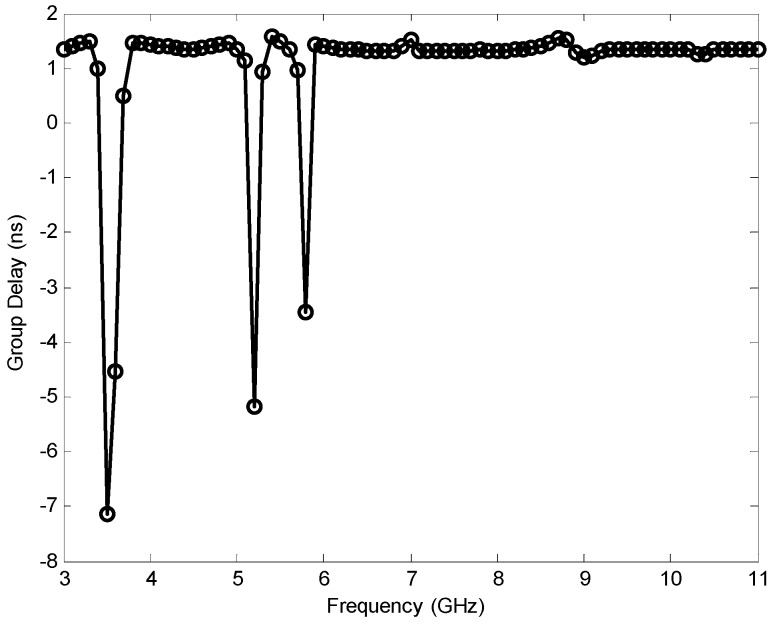
Group delay of the proposed antenna.

The group delay is defined as the first derivative of the far field phase of the transmission response with respect to the radial frequency ω [40]. The measured group delay is shown in Figure 15. Three sharp variations (about 8.5, 6.5, and 4.8 ns) of the group delay in the notched bands at 3.5, 5.2, and 5.8 GHz were observed, which indicates the obvious deviation from linear phase response. These sharp variations can be interpreted from the VSWR results because serious impedance mismatch in these frequencies leads to deteriorated transmission response including sharply reduced magnitude and nonlinear phase distortions. At other frequencies outside the notched bands, the group delay remains nearly constant, which indicates good phase linearity.

**Figure 16:**
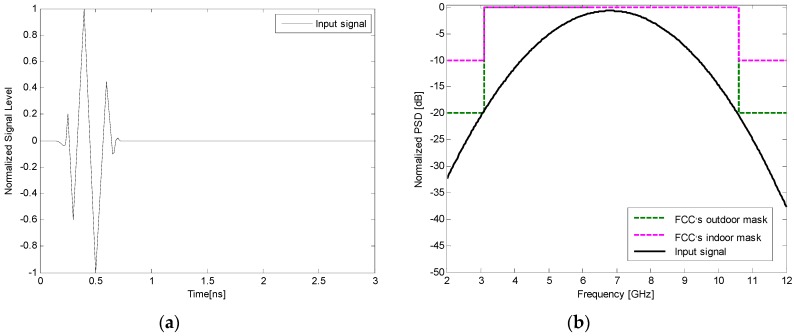
(**a**) Normalized input pulse; (**b**) power spectrum density and Federal Communication Commission (FCC) mask for indoor and outdoor mask.

**Figure 17:**
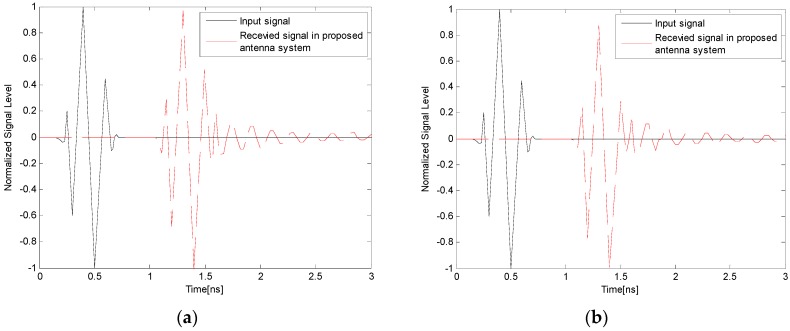
Input and received pulse waveforms of the proposed antenna: (**a**) in face-to-face scenario; and (**b**) in side-by-side scenario.

To further assess the suitability of the proposed antenna for time-domain pulse transmission, two transmitting and receiving antenna systems including face-to-face and side-by-side scenarios were investigated. In both scenarios, two identical proposed antennas placed at a distance of 20 cm from each other were employed for transmitting and receiving the UWB signal. An input pulse with a frequency spectrum corresponding to 3.1–10.6 GHz was used as the input signal, as shown in Figure 16. The power spectral density (PSD) of the input pulse conforms well to the indoor and outdoor PSD mask defined by the FCC. Figure 17 shows the input and received signals of the proposed antenna system in face-to-face and side-by-side scenarios, respectively. It is observed from the figure that the received signals of the proposed antenna in both scenarios have similar waveforms with small distortion. To quantitatively measure the similarity between the input and received signals, a key factor known as the fidelity factor (FF) [41] is also calculated. Usually, a distortion higher than 50% (FF < 0.5) may cause the pulse to become almost unrecognizable [41]. The calculated fidelity factors were 86% and 81% when the proposed antenna system was configured face to face and side by side, respectively. The results indicate that the proposed antenna has good potential in transmitting UWB impulse signals with tolerable distortion.

In summary, a nearly constant group delay, a low-variation transmission coefficient, and a good fidelity factor show that the proposed antenna exhibits good phase linearity at desired UWB frequencies and hence good pulse-preserving capabilities as demanded by short distance pulse-based UWB communications.

### 3.3. Comparison with Other Reported Designs

To validate the effectiveness of our design, Table 2 presents the performance comparison of our proposed triple band-notched UWB antenna with other recently reported antennas, including size, impedance bandwidth, notched bands, and maximum VSWR values in the notched bands. It can be concluded from the table that, compared to other recently reported antennas [18,19,20,21,22,23,24,25,26,27,28,29,30,31,32,33,34,35,36], our proposed antenna has some obvious advantages such as a much smaller size, a wider impedance bandwidth, and more complete rejecting bands. Note that our design consists of regular symmetrical slotted patches only and is easy to replicate, fine-tune, and fabricate, while most of the antennas mentioned in the table employ irregular-shaped patches and parasitic stubs/resonators as well as etching various irregular-shaped slots on the patch or ground, which leads to a complex overall structure and the fabrication difficulty. In addition, the designs in [19,20,21,22,23,24,25,26,27,28,29] completely reject the entire 5–6 GHz frequency band. Hence, any useful information within the frequency band of 5.35–5.725 GHz will be lost, meaning the degraded receive information and thus a lower signal quality [30].

## 4. Conclusions 

In this paper, a compact planar printed monopole antenna with 3.5/5.2/5.8 GHz triple band-notched characteristics and broad bandwidth characteristic for UWB applications is proposed. The antenna can operate from 2.12 GHz to 14.80 GHz with triple-notched bands of 3.36–4.16, 4.92–5.36, and 5.68–6.0 GHz for rejecting the 3.5 GHz WiMAX and 5.2/5.8 GHz WLAN interferences. By etching several slots with various shapes and dimensions on the radiation patch, triple band-notched properties are generated, and by integrating the slotted radiating patch with a rectangular ground plane on the back of the substrate, improved impedance bandwidth can be achieved, especially at the higher band. The designed antenna has a simple symmetrical linear structure and a very compact size of 10 × 16 mm^2^, such that it is easily embedded within portable IoT sensors. The proposed antenna provides good frequency-domain and time-domain performances, such as nearly omnidirectional radiation characteristics, a sharply decreased gain at the stopbands, a flat group delay, and low pulse distortion, which make it suitable for short-range pulse-based UWB communications.

## Figures and Tables

**Table 1:** Optimal dimensions of the proposed antenna.

Parameter	Value (mm)	Parameter	Value (mm)
*l*	16	*w*	10
*l*_1_	9	*w*_1_	9
*l*_2_	6	*w*_2_	2
*l*_3_	0.5	*w*_3_	3
*l*_4_	0.5	*w*_4_	1.5
*l*_5_	7	*w*_5_	0.5
*l*_6_	6.5	*w*_6_	1.5
*l*_7_	4	*w*_7_	0.5
*l*_8_	1.5	*w*_8_	0.5
*l*_9_	1.5	*w*_9_	0.5
*l*_10_	3.5	*w*_10_	1
*l*_11_	1.5	*w*_11_	0.5

**Table 2:** Performance comparison of the proposed triple band-notched UWB antenna with other reported antennas.

References	Size (mm)	Bandwidth (GHz)	Notched Band (GHz)	VSWR_max_ *	Remarks
[18]	24 × 28	3–13 (125%)	5.09–5.36, 5.65–5.9	5.0, 4.5	Large overall size and only two notched bands
[19]	26 × 30	2.5–25 (164%)	3–3.8, 5.1–6.2	6.7, 5.5	Large overall size and only two notched bands
[20]	14 × 16	3.2–11 (110%)	3.3–4.2, 5.0–6.0	5.8, 4.4	Large overall size and Only two notched bands
[21]	40.4 × 44	3–11 (114%)	5.15–5.35, 5.725–5.825	6.73, 6.1	Large overall size and Only two notched bands
[22]	35 × 30	3–11 (114%)	4.91–5.9	3.6	Large overall size and Only one desirable notched band
[23]	22 × 8.5	3.2–10.6 (107%)	5.15–5.85	7.0	Only one desirable notched band
[24]	36 × 33	3.1–22 (151%)	5.1–5.9	3.6	Large overall size and Only one desirable notched band
[25]	20 × 27	2.89–11.52 (120%)	3.18–3.85, 5.0–6.0	8.3, 9.2	Large overall size and only two notched bands
[26]	20 × 25	2.7–14 (135%)	3.3–3.8, 5.0–6.1	4.8, 2.8	Large overall size and only two notched bands
[27]	35 × 30	2.9–10 (110%)	3.3–4.2, 5.2–5.9	6.8, 6.2	Large overall size and only two notched bands
[28]	30 × 30	2.7–14.4 (137%)	3.0–3.9, 4.9–5.8	7.6, 6.5	Large overall size, complicated irregular structure, and only two notched bands
[29]	25 × 20	2.85–12 (123%)	3.3–3.8, 5.15–5.85	5.4, 6.4	Large overall size, complicated irregular structure, and only two notched bands
[30]	36 × 34	2.9–13 (127%)	3.3–3.9, 5.2–5.35, 5.8–6.0	8.0, 5.5, 6.4	Large size, complicated irregular structure, incomplete rejecting for 5.15–5.35 and 5.725–5.825 bands
[31]	24 × 34.6	3.1–11 (112%)	3.4–3.6, 5.1–5.3, 5.7–5.9	4.4, 3.0, 3.6	Large overall size, complicated irregular structure, incomplete rejecting for 3.3–3.69 and 5.15–5.35 bands
[32]	24 × 30	2.6–12 (129%)	3.3–4, 5.15–5.4, 5.8–6.1	5.0, 3.5, 3.0	Large overall size , complicated irregular structure, incomplete rejecting for 5.725–5.825 bands
[33]	26 × 31.8	2.8–12.6 (127%)	3.43–3.65, 4.95–5.25, 5.36–5.85	5.0, 5.2, 3.5	Large overall size, incomplete rejecting for 3.3–3.69 and 5.15–5.35 bands
[34]	22.5 × 24	3.2–11.6 (111%)	3.20–4.19, 5.02–5.32, 5.51–6.10	14.0, 4.0, 5.5	Large overall size, complicated irregular structure, incomplete rejecting for 5.15–5.35 band
[35]	25 × 30	3.02–11.1 (114%)	3.25–3.6, 5.0–5.4, 5.7–6.1	8.7, 6.3, 4.8	Large overall size, some irregular structures, incomplete rejecting for 3.3–3.69 band
[36]	27 × 34	3–10.6 (112%)	3.29–3.67, 5.12–5.35, 5.67–5.83	4.5, 5.9, 2.8	Large overall size, some irregular structures
Proposed antenna	10 × 16	2.12–14.80 (150%)	3.36–4.16, 4.92–5.36, 5.68–6.0	15.1, 4.0, 3.0	Wider operation bandwidth, compact size, sufficient and complete band-notched function

* VSWR_max_ denotes the maximum VSWR levels in the notched bands.

## References

[1] The Federal Communications Commission (2002). Revision of Part 15 of the Commission’s Rules Regarding Ultra Wideband Transmission Systems.

[2] The Federal Communications Commission (2007). Revision of Part 15 of the Commission’s Rules Regarding Ultra Wideband Transmission Systems.

[3] Chóliz J., Hernández Á., Valdovinos A. (2011). A Framework for UWB-Based Communication and Location Tracking Systems for Wireless Sensor Networks. Sensors.

[4] Zhang J., Orlik P.V., Sahinoglu Z., Molisch A.F., Kinney P. (2009). UWB Systems for Wireless Sensor Networks. Proc. IEEE.

[5] Valderas D., Sancho J.I., Puente D., Ling C., Chen X. (2010). Ultrawideband Antennas: Design and Applications.

[6] Dumoulin A., John M., Ammann M., McEvoy P. (2012). Optimized monopole and dipole antennas for UWB asset tag location systems. IEEE Trans. Antennas Propag..

[7] Li X., Bond E.J., Van Veen B., Hagness S. (2005). An overview of ultra-wideband microwave imaging via space-time beamforming for early-stage breast-cancer detection. IEEE Antennas Propag. Mag..

[8] Islam M.T., Islam M.M., Samsuzzaman M., Faruque M.R.I., Misran N. (2015). A Negative Index Metamaterial-Inspired UWB Antenna with an Integration of Complementary SRR and CLS Unit Cells for Microwave Imaging Sensor Applications. Sensors.

[9] Koohestani M., Moreira A.A., Skrivervik A.K. (2015). System fidelity factor evaluation of wearable ultra-wideband antennas for on-body communications. IET Microw. Antennas Propag..

[10] Ning H. (2013). Unit and Ubiquitous Internet of Things.

[11] Chahat N., Zhadobov M., Sauleau R., Ito K. (2011). A compact UWB antenna for on-body applications. IEEE Trans. Antennas Propag..

[12] Guo L., Wang S., Chen X., Parini C.G. (2010). Study of compact antenna for UWB applications. Electron. Lett..

[13] Bekasiewic A., Koziel S. (2016). Compact UWB monopole antenna for internet of things applications. Electron. Lett..

[14] Yang J., Wang H., Lv Z., Wang H. (2016). Design of miniaturized dual-band microstrip antenna for WLAN application. Sensors.

[15] Rotaru M., Sykulski J. (2010). Compact Electromagnetic Bandgap Structures for Notch Band in Ultra-Wideband Applications. Sensors.

[16] Kerkhoff A., Ling H. Design of a planar monopole antenna for use with ultra-wideband (UWB) having a band-notched characteristic. Proceedings of the 2003 IEEE Antennas and Propagation Society International Symposium.

[17] Schantz H.G., Wolence G., Myszka E.M. Frequency notched UWB antenna. Proceedings of the IEEE Conference on Ultra-Wideband Systems and Technologies.

[18] Li T., Zhai H., Li G., Li L., Liang C. (2012). Compact UWB band-notched antenna design using interdigital capacitance loading loop resonator. IEEE Antennas Wirel. Propag. Lett..

[19] Emadian S.R., Ghobadi C., Nourinia J., Mirmozafari M.H., Pourahmadazar J. (2012). Bandwidth enhancement of CPW-fed circle-like slot antenna with dual band-notched characteristic. IEEE Antennas Wirel. Propag. Lett..

[20] Lotfi P., Azarmanesh M., Soltani S. (2013). Rotatable dual band-notched UWB/triple-band WLAN reconfigurable antenna. IEEE Antennas Wirel. Propag. Lett..

[21] Gheethan A.A., Anagnostou D.E. (2012). Dual band-reject UWB antenna with sharp rejection of narrow and closely-Spaced bands. IEEE Trans. Antennas Propag..

[22] Li B., Hong J., Wang B. (2012). Switched band-notched UWB/dual-band WLAN slot antenna with inverted S-shaped slots. IEEE Antennas Wirel. Propag. Lett..

[23] Chu Q., Mao C., He Z. (2013). A compact notched band UWB slot antenna with sharp selectivity and controllable bandwidth. IEEE Trans. Antennas Propag..

[24] Tang Z.J., Wu X.F., Zhan J. (2015). Novel compact band-notched UWB antenna using convex-shaped slot patch. Microw. Opt. Technol. Lett..

[25] Gao P., Xiong L., Dai J., He S., Zheng Y. (2013). Compact printed wide-slot UWB antenna with 3.5/5.5-GHz dual band-notched characteristics. IEEE Antennas Wirel. Propag. Lett..

[26] Ali M.M., Saad A.R., Khaled E.M. (2013). A design of miniaturized ultra-wideband printed slot antenna with 3.5/5.5 GHz band-notched characteristics: Analysis and implementation. Prog. Electromagn. Res. B.

[27] Shi R., Xu X., Dong J., Luo Q. (2014). Design and analysis of a novel dual band-notched UWB antenna. Int. J. Antennas Propag..

[28] Yadav S., Gautam A.K., Kanaujia B.K. (2015). Design of dual band-notched lamp-shaped antenna with UWB characteristics. Int. J. Microw. Wirel. Technol..

[29] Srivastava G., Mohan A. (2015). A planar UWB monopole antenna with dual band notched function. Microw. Opt. Technol. Lett..

[30] Li W.T., Shi X.W., Hei Y.Q. (2009). Novel Planar UWB monopole antenna with triple band-notched characteristic. IEEE Antennas Wirel. Propag. Lett..

[31] Sarkar M., Dwari S. (2015). Printed monopole antenna for ultra-wideband application with tunable triple band-notched characteristics. Wirel. Pers. Commun..

[32] Liao X.J., Yang H.C., Han N., Li Y. (2011). Aperture UWB antenna with triple band-notched characteristic. Electron. Lett..

[33] Xu J., Wang G. (2012). A compact printed UWB antenna with triple band-notched characteristics. Microw. Opt. Technol. Lett..

[34] Ali M.M., Saad A.R., Khaled E.M. (2014). Implementation and justification of a triple frequency-notched UWB proximity-fed antenna with shunt stubs. Microw. Opt. Technol. Lett..

[35] Tang M.-C., Xiao S., Deng T., Wang D., Guan J., Wang B., Ge G.-D. (2011). Compact UWB antenna with multiple band-notches for WiMAX and WLAN. IEEE Trans. Antennas Propag..

[36] Lin C.-C., Jin P., Ziolkowski R.W. (2012). Single, Dual and Tri-Band-Notched Ultrawideband (UWB) Antennas Using Capacitively Loaded Loop (CLL) Resonators. IEEE Trans. Antennas Propag..

[37] Janaswamy R., Schaubert D.H. (1986). Characteristic impedance of a wide slotline on low-permittivity substrates. IEEE Trans. Microw. Theory Technol..

[38] Gupta K., Garg R., Bahl I., Bhartis P. (1996). Microstrip Lines and Slotlines.

[39] Taylor J.D. (1995). Introduction to Ultra-Wideband Radar System.

[40] Cho Y.J., Kim K.K., Choi D.H., Lee S.S., Park S.O. (2006). A miniature UWB planar monopole antenna with 5 GHz band rejection filter and the time domain characteristics. IEEE Trans. Antennas Propag..

[41] Quintero G., Zürcher J.F., Skrivervik A.K. (2011). System fidelity factor: A new method for comparing UWB antennas. IEEE Trans. Antennas Propag..

